# Efficacy of anti-ICOS agonist monoclonal antibodies in preclinical tumor models provides a rationale for clinical development as cancer immunotherapeutics

**DOI:** 10.1186/2051-1426-3-S2-O9

**Published:** 2015-11-04

**Authors:** Christopher Harvey, Kutlu Elpek, Ellen Duong, Tyler Simpson, ChengYi J Shu, Lindsey Shallberg, Matthew Wallace, Sriram Sathyanarayanan, Robert Mabry, Michael Briskin, Jennifer Michaelson, Thomas F Gajewski

**Affiliations:** 1 Jounce Therapeutics, Cambridge, MA, USA; 2 University of Chicago, Chicago, IL, USA

## 

ICOS (inducible co-stimulator molecule) is a T cell encoded member of the extended B7/CD28 superfamily that is up-regulated upon early T cell activation. Previous studies have shown that higher ICOS expression on circulating T cells, post treatment with ipilimumab, is associated with better clinical outcome. In complementary pre-clinical studies, efficacy observed with a whole cell vaccine consisting of ICOS-L expressing B16 melanoma (IVAX) suggests that agonism of this pathway could provide therapeutic benefit in the cancer setting.

We have generated a panel of anti-ICOS monoclonal antibodies with in vitro agonist properties. These lead agonist mAbs have been shown to be efficacious both as monotherapies in multiple syngeneic tumor models and in combination with an anti-PD1 antibody. Mechanistic studies that demonstrate tumor regression is associated with enhanced ratios of cytotoxic CD8/regulatory T (Tregs) cells as well as preferential reduction in ICOS high Tregs in the tumor microenvironment have informed the selection of optimal Fc regions for clinical development.

Jounce is now developing a high affinity humanized agonist monoclonal antibody to be tested as both a monotherapy as well as in combination with other T cell checkpoints in solid tumor indications.

